# LncRNA GAS5 inhibits Invasion and Migration of Lung Cancer through influencing EMT process

**DOI:** 10.7150/jca.56218

**Published:** 2021-04-02

**Authors:** Lihuan Zhu, Dongsheng Zhou, Tianxing Guo, Wenshu Chen, Yun Ding, Wujing Li, Yangyun Huang, Jianyuan Huang, Xiaojie Pan

**Affiliations:** 1 Department of Thoracic Surgery, Fujian Provincial Hospital, Fuzhou, China.; 2 Department of Radiology, Fujian Provincial Hospital, Fuzhou, China.

**Keywords:** GAS5, lung cancer, EMT, E-cadherin

## Abstract

**Background:** Lung cancer is a malignant tumor in mammary gland epithelium with high morbidity and mortality among women worldwide. Long noncoding RNA GAS5 (GAS5) has been proved to be closely related with tumor progression. However, the influence of GAS5 on lung cancer and the specific mechanism remain unclear.

**Methods:** Cell invasion, cell migration, cell apoptosis and cell cycle were investigated after transfection with pcDNA-GAS5 and sh-GAS5. Sizes of tumors were measured by establishing transplanted tumor model *in vivo*. E-cadherin and N-cadherin expressions were investigated.

**Results:** Cell invasion and migration were inhibited markedly in GAS5 overexpressed cell line. Cell cycle results indicated that the percentage of S-phase cells was increased, and G2-phase was reduced in the GAS5 overexpression cell line. Tumor size was suppressed obviously after GAS5 overexpression treatment. GAS5 markedly inhibited the expression of E-cadherin and induced the expression of N-cadherin. GAS5 overexpression significantly inhibited lung cancer cell proliferation by increasing the E-cadherin and decreasing N-cadherin.

**Conclusions:** These findings provide novel evidence that GAS5 can be viewed as an anti-lung cancer agent through affecting EMT pathway.

## Introduction

Lung cancer is one of the most common cancers worldwide, and characterized with high mortality. Nowadays, chemotherapy and surgery have been greatly improved, but the 5-year survival rate of lung cancer patients remains very low 1. Traditional chemoradiotherapy has been widely used for the treatment of lung cancer patients. However, chemoradiotherapy could also kill normal cells. In addition, myelosuppression, gastrointestinal dysfunction, alopecia, and dysfunction of immune system are also common side effects.

LncRNAs are long non-coding single stranded RNAs with more than 200 nucleotides, play various roles in regulating chromatin dynamics, gene expression, growth, differentiation and development 2. Genome-wide association research on tumor samples have identified a large number of lncRNAs associated with multiple types of cancer 3. Many lncRNAs rooted in the nucleus where they actively interact with chromatin remodeling complexes to regulate the expression of genes 4, 5. They may have an impact on cell proliferation and act as an oncogene or putative therapeutic target.

Growth arrest-special transcript 5 (GAS5), encodes 1q25, a chromosomal locus associated with lymphoma. GAS5 was believed to be a tumor suppressor in colorectal cancer 6. Qiao et al. found that decreased GAS5 was closely linked with renal cell carcinoma, but overexpression of GAS5 can suppress renal cell carcinoma. Fujun et al. found that GAS5 was down-regulated in most hepatic carcinoma patients, and the GAS5 was an independent prognostic factor for liver cancer patients 7. In addition, GAS5 expression level was significantly lower in bladder cancer patients. Gastric cancer cell proliferation was inhibited by GAS5 through regulating CDK6 8, 9. In addition, GAS5 expression was down-regulated compared to normal breast epithelial tissue 10. The amount of GAS5 in serum was inversely related to postoperative lymph node metastasis status 11.

Epithelial-mesenchymal transition (EMT) is a process that epithelial cells acquire mesenchymal features. It was usually observed under physiological conditions such as embryogenesis and wound healing. Meanwhile, it can also be observed in some pathological states including organ fibrosis and cancer genesis. EMT is characterized by the features that epithelial tumor cells lose their epithelial phenotype and obtain the ability to migrate and promote metastasis 12. EMT could accelerate metastasis, and cells with different EMT levels present different metastatic properties 13, 14, and the specific mechanism needs to be elucidated.

It has been increasingly recognized that drug resistance is frequently accompanied with EMT in various cancers, including pancreatic cancer 15, lung cancer 16 and bladder cancer 17. In this study, we aimed to clarify the correlation between GAS5 and lung cancer cells. Meanwhile, the potential role of GAS5 as an anti-cancer agent could be concluded in this study.

## Materials and Methods

### Cell culture and cell line construction

Human lung cancer cell line H-460, H-129 and A-549 were used in this study. All cells were cultured with DMEM medium containing 10% fetal bovine serum (FBS, #16140071, Hyclone) and 1% penicillin-streptomycin in a cell incubator at 37 °C 5% CO_2._ 0.25% trypsin-EDTA was added to digest cells. All experimental cells were in the logarithmic growth phase.

The cells were seeded into 6-well plates and transfected with sh-GAS5 or GAS5 overexpression vectors (pcDNA3.1-GAS5), respectively. sh-GAS5 interference fragment (20 μM, 5 μL) or recombinant plasmid pcDNA3.1-GAS5 (4 μg) was diluted in 100 μl serum-free medium. 10 μL Lipofectamine 2000 (#11668019, Invitrogen) was diluted in 100 μl serum-free medium, and the diluted interfering fragments or plasmids were mixed with diluted transfection reagent and incubated for 20 min at room temperature. The transfection mixture was slowly added to the plate containing 2.0 mL serum-free medium per well and replaced with complete medium containing 10% fetal bovine serum. Details about culturing and selecting the silencing GAS5 expression cell lines were described previously 18.

### Transwell assays (cell migration and invasion assay)

Cells were digested with trypsin at logarithmic growth stage. The cells were prepared into single-cell suspension and seeded into a 6-well plate. When cell confluence reached about 80%, a wound was drawn with a pipette tip. The width of each cell wound was observed after 48 h.

Digested cells were seeded on the top chamber of the Transwell plates. 100 μL cell suspension was added to the upper chamber, and DMEM medium containing 10% FBS was added to the lower chamber. After culturing for 24 h at 37 °C and 5% CO_2_, cells were fixed with 4% polymethanol, and stained with Giemsa. The number of cells passed through was observed and photographed under high magnification microscope.

### Flow cytometry analysis

Flow cytometry was conducted as described previously 19. Cells were prepared into 6-well plates and cultured in an incubator overnight. Cells were digested, and centrifuged at 500×g to collect the cell pellet. Then, cells were treated with Annexin V-FITC and propidium iodide (PI) (#40302ES20, Yeasen Biotech Co., Ltd., China). After incubation for 15 min at room temperature in the dark, apoptosis was analyzed through flow cytometry.

### RNA purification and quantitative RT-PCR (qRT-PCR)

#### RNA purification

Cells was subjected to extract total RNA using TRIzol Reagent (#15596026, Thermo Fisher) according to the manufacturer's protocol. Proteinase K and RNase free DNase I was used to remove any residual protein or DNA. The purity of the RNA was measured using NanoDrop 8000 (Thermo) by detecting the OD 260 nm/280 nm value. The OD 260 nm/280 nm value was 1.96.

#### qRT-PCR

1 ng RNA was added to the quantitative one step RT-PCR reaction system (#SD103, Tiangen Biotech Co., LTD. China) including RTase (0.4 μL), Taq polymerase (2.5 μL) and mixture buffer. The qRT-PCR reaction steps were listed as follows: 50 °C for 30 min, initial template predenaturation 95 °C for 2 min, 40 cycles of denaturation 94 °C for 20s, annealing 55 °C for 60s and extension 68 °C 20s. Data was analyzed by normalizing expression of target genes to glyceraldehyde-3-phosphate dehydrogenase (GAPDH) using ABI 7500 Real-Time PCR System. The mRNA expression between treatment group and control group were calculated using the 2^-ΔΔCt^ method.

### Western blot

Polyclonal antibodies anti-E-cadherin (#ab40772, abcam), anti-N-cadherin (#ab76011, abcam), actin reference antibody (#ab5694, abcam), and second antibody (goat anti-rabbit lgG, #ab205718) were purchased from Abcam. Western blotting was carried out following standard procedures described previously 18.

### Immunohistochemistry experiment

After sacrificing mice, tumors were harvested and the weight was measured. Then tumor tissues were immersed using neutral formalin for fixation for 24 h. All tumor tissues and one lung tissue in each group were paraffin-embedded, 4 μm thickness continuous sections were made. Gradient dehydration with xylene and alcohol, hematoxylin staining, 1% hydrochloric acid alcohol differentiation, warm water cyanidation, eosin restaining, dehydration, transparent and neutral gum sealing tablets, specific experimental steps are conducted 10. For immunohistochemistry analysis, the corresponding primary antibody was used to incubate with the sectioned tissue. The antibody details were listed as follows: anti-E-cadherin (#ab40772, abcam), anti-N-cadherin (#ab76011, abcam).

### Establishment of metastatic tumor model in mice

Female nude mice (C57BL/6, 5-weeks old) were purchased from Beijing Vital River Laboratory Animal Technology (Beijing, China). The animal experiments were approved by the Institutional Animal Care of Fujian Provincial Hospital (Approval number: #2020018). GAS5 gene silence cells (sh-GAS5 group) and overexpression cells (pcDNA3.1 GAS5) were inoculated into the right side of the pad, PBS group was used as blank control group, and each group included 4 mice. When the tumor grew to around 7 mm×7 mm×7 mm, mice were anesthetized with ketamine, and sacrificed by neck amputation. Tumors were harvested to measure the size and weight of the tumor was analyzed by subsequent immunohistochemistry.

### Effect of GAS5 on chemotherapy sensitivity of lung cancer cells

Three common chemotherapy drugs (fluorouracial, doxorubicin, and cisplatin) were used. Overexpression or knockdown of GAS in H-460 cells were established as described above. Then, cells were treated with different concentrations of fluorouracial (0, 40, 80, and 120 g/mL), doxorubicin (0, 1, 5, and 10 µM), or cisplatin (0, 10, 30, and 50 µM) for 24 h. Then, the cell proliferation was measured using MTT assay.

### Statistical analysis

Data was showed as mean values with standard deviation. Student's t test was used to analyze data between 2 groups. Data among more than 2 groups was analyzed using one-way analysis of variance (ANOVA). P value < 0.05 was considered as statistically significant. All results were achieved by performing at least triple independent experiments.

## Results

### Expression of GAS5 *in vitro* and *in vivo*

**Figure 1:**
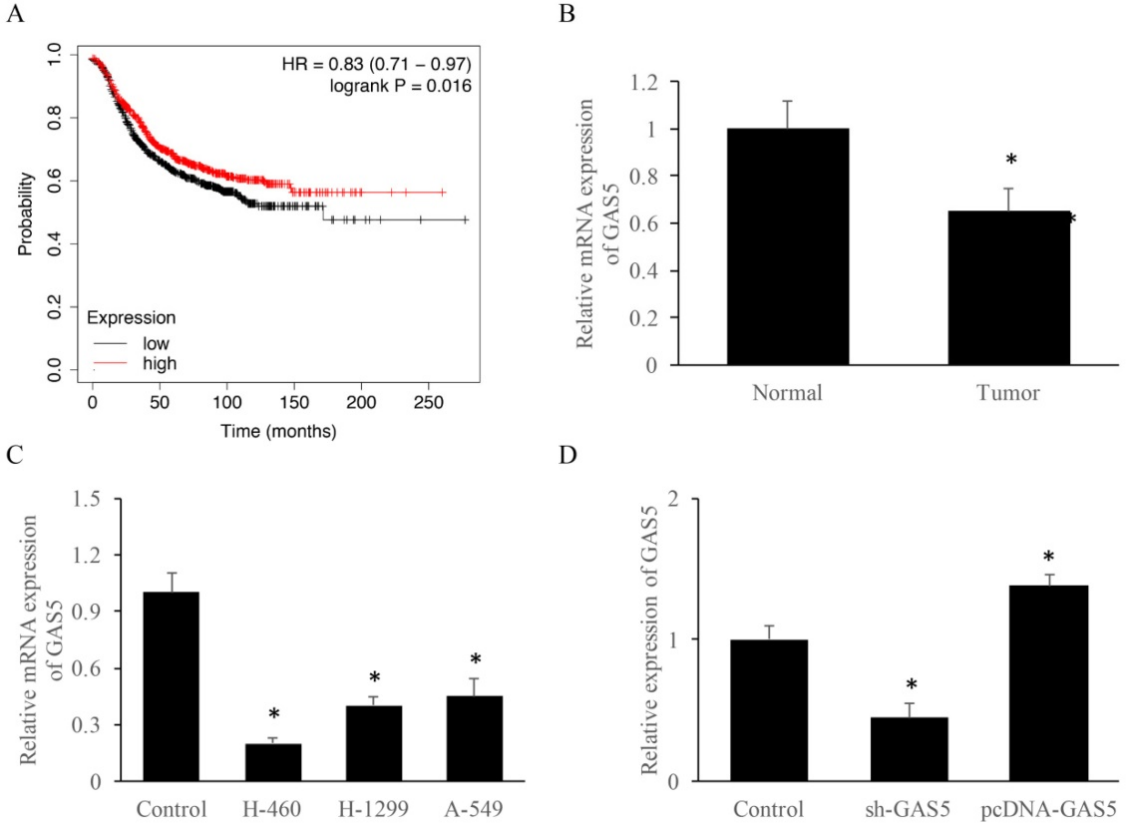
LncRNA GAS5 expression among lung cancer patients and different lung cancer cell lines. (A) Relationship between GAS5 expression and patient survival; (B) quantification analysis of GAS5 expression in lung cancer tissues; (C) quantification analysis of GAS5 expression level in three lung cancer cell lines; (D) quantification detection of GAS5 knockdown (sh-GAS5) and overexpression (pcDNA-GAS5) cell lines. * P<0.05 compared with group Control. The results were achieved by conducting at least 3 independent experiments.

To explore the correlation between survival rate and GAS5 expression level, Kmplot database (https://kmplot.com/analysis/) was used. Results indicated that those who highly expressed GAS5 have a 10% higher survival rate (Figure 1A). RT-PCR data suggested that GAS5 expression was reduced about 2-folds in lung cancer tissue than that of the normal group (Figure 1B). Furthermore, GAS5 expression of different lung cancer cell lines (H-460, H-129 and A-549) was evaluated, respectively. Results showed that the expression of GAS5 in H-460 was lower than other two cell types, thus H-460 was selected for subsequent experiments (Figure 1C). Moreover, cell models of GAS5 knockdown (Sh-GAS5) or overexpression (pcDNA-GAS5) was established (Figure 1D).

### sh-GAS5 markedly promoted while pcDNA-GAS5 significantly inhibited cell migration and invasion

**Figure 2:**
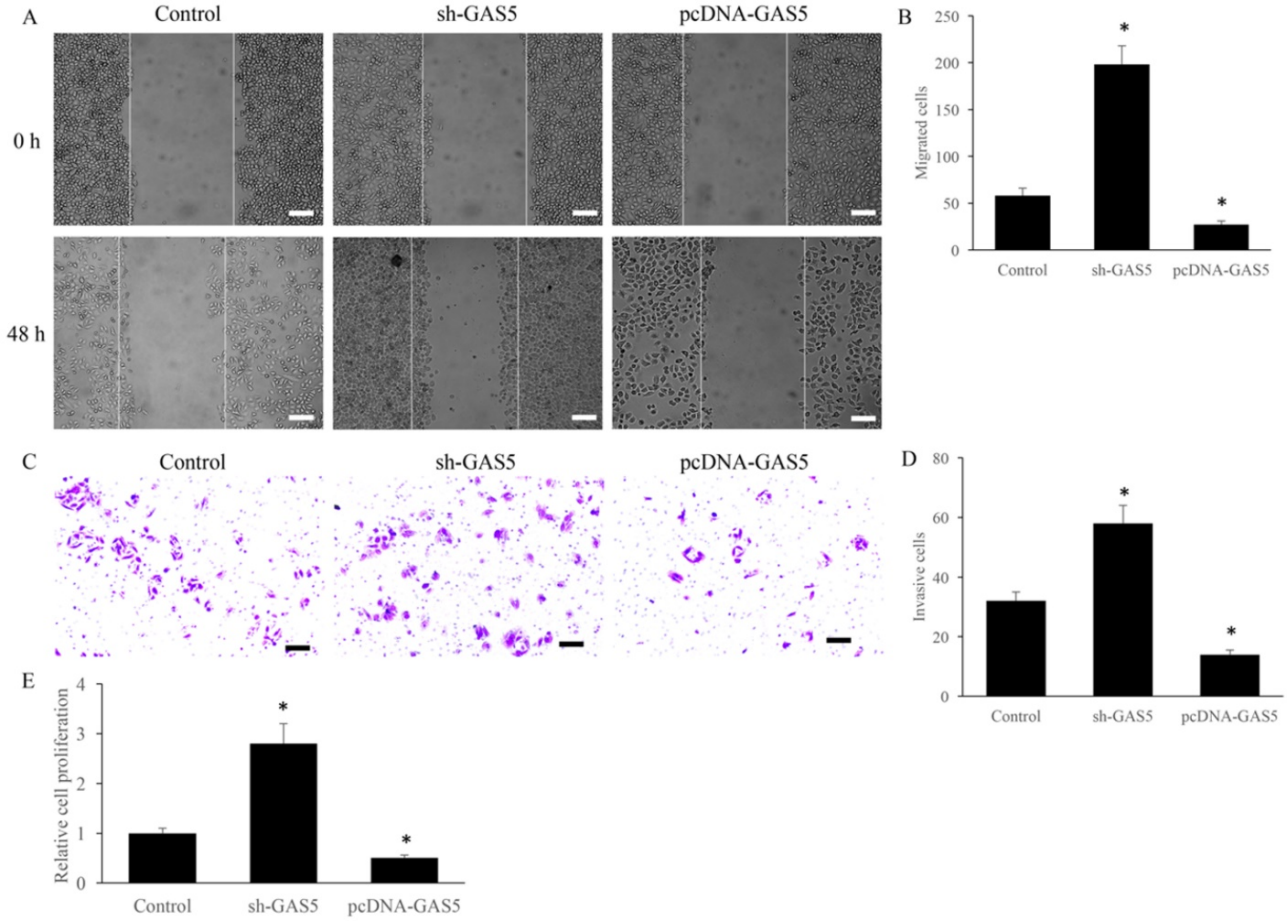
GAS5 markedly inhibited the migration and invasion of lung cancer cells. (A) Cell migration of GAS5 knockdown (SH-GAS5) and overexpression (PCDNA-GAS5) cell lines, scale bar: 300 µm; (B) quantification analysis of cell migration among SH-GAS5 and PCDNA-GAS5 cell lines; (C) Cell invasion of SH-GAS5 and PCDNA-GAS5 cell lines, scale bar: 50 µm; (D) quantification analysis of SH-GAS5 and PCDNA-GAS5 cell lines; (E): Influence of SH-GAS5 and PCDNA-GAS5 on cell proliferation. * P<0.05 compared with group Control. The results were achieved by conducting at least 3 independent experiments.

Cell migration and invasion have been believed to be closely related to tumor metastasis. The effect of GAS5 on the migration and invasion were investigated using transwell assay. sh-GAS5 promoted the migration and invasion of H-460 cell, and overexpression of GAS5 significantly inhibited the migration and invasion (Figure 2A-D). Further, study *in vitro* showed that sh-GAS5 markedly increased cell proliferation rate (Figure 2E).

### GAS5 significantly increased the proportion of S phase cells and reduced the proportion of G2 phase cells

**Figure 3:**
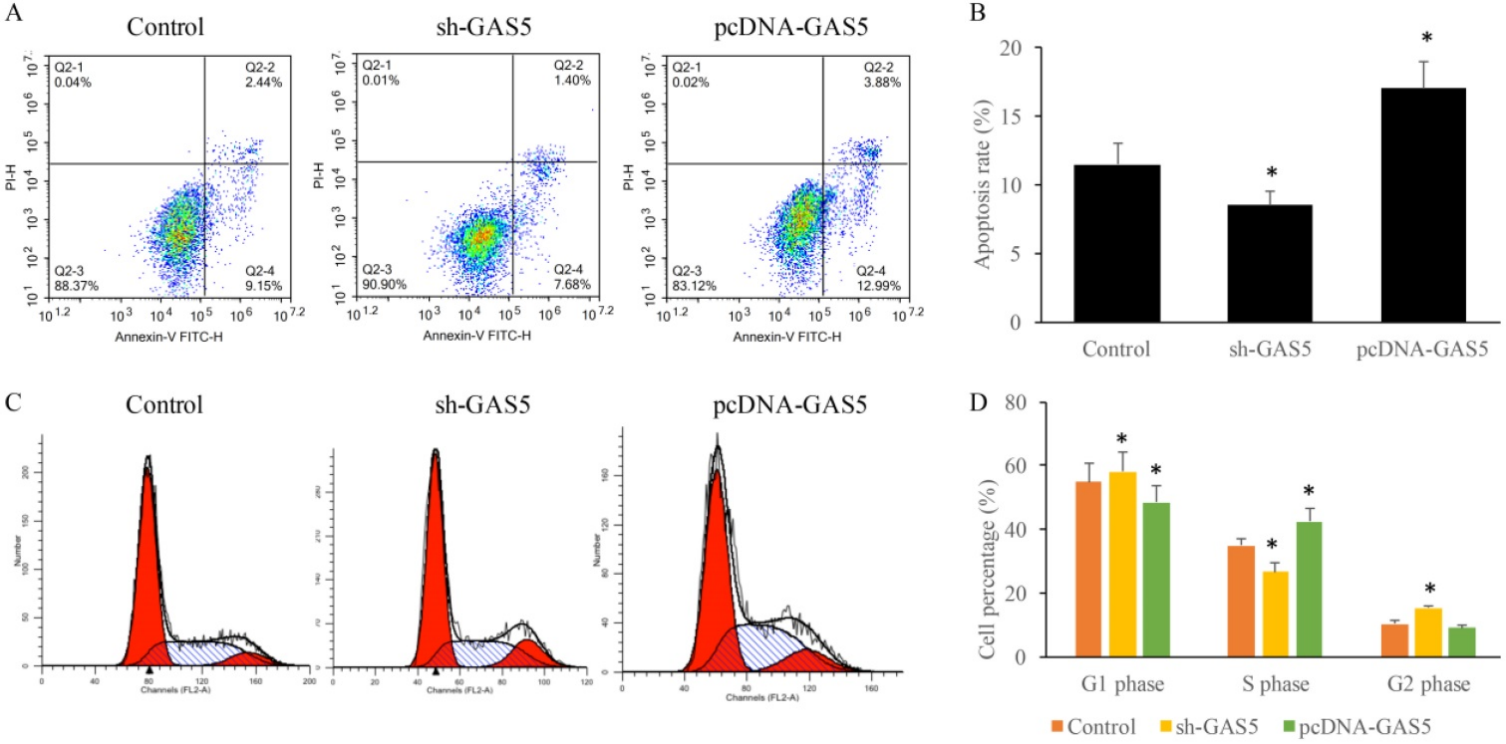
GAS5 markedly induced cell apoptosis of human lung cancer cells using flow cytometry analysis and significantly decreased the percentage in G2 stage and increased in S stage and of the cell cycle. (A) Cell apoptosis was measured of SH-GAS5 and PCDNA-GAS5 cell lines; (B) quantification analysis of cell apoptosis among SH-GAS5 and PCDNA-GAS5 cell lines; (C) Representative picture of cell cycle among SH-GAS5 and PCDNA-GAS5 cell lines; (D) quantification analysis of cells in S stage and G2 stage among SH-GAS5 and PCDNA-GAS5 cell lines. * P<0.05 compared with group Control. The results were achieved by conducting at least 3 independent experiments.

To determine the influence of GAS5 on lung cancer cell apoptosis and cell cycle, cells treated with sh-GAS5 and pcDNA-GAS5 was detected with flow cytometer. Results revealed that sh-GAS5 inhibited cell apoptosis, and pcDNA-GAS5 promoted apoptosis (Figure 3A-B). Cell cycle results indicated total S-phase was 26.16% and G2 phase was 16.18% in the sh-GAS5 group, S-phase and G2 phase in the control group was 32.88% and 8.04%. The percentage of S-phase cells was increased, G2-phase was reduced in overexpressed cell lines. The results proved that GAS5 play a vital role in the cell division, proliferation and survival.

### GAS5 induced E-cadherin expression and inhibited N-cadherin expression

**Figure 4:**
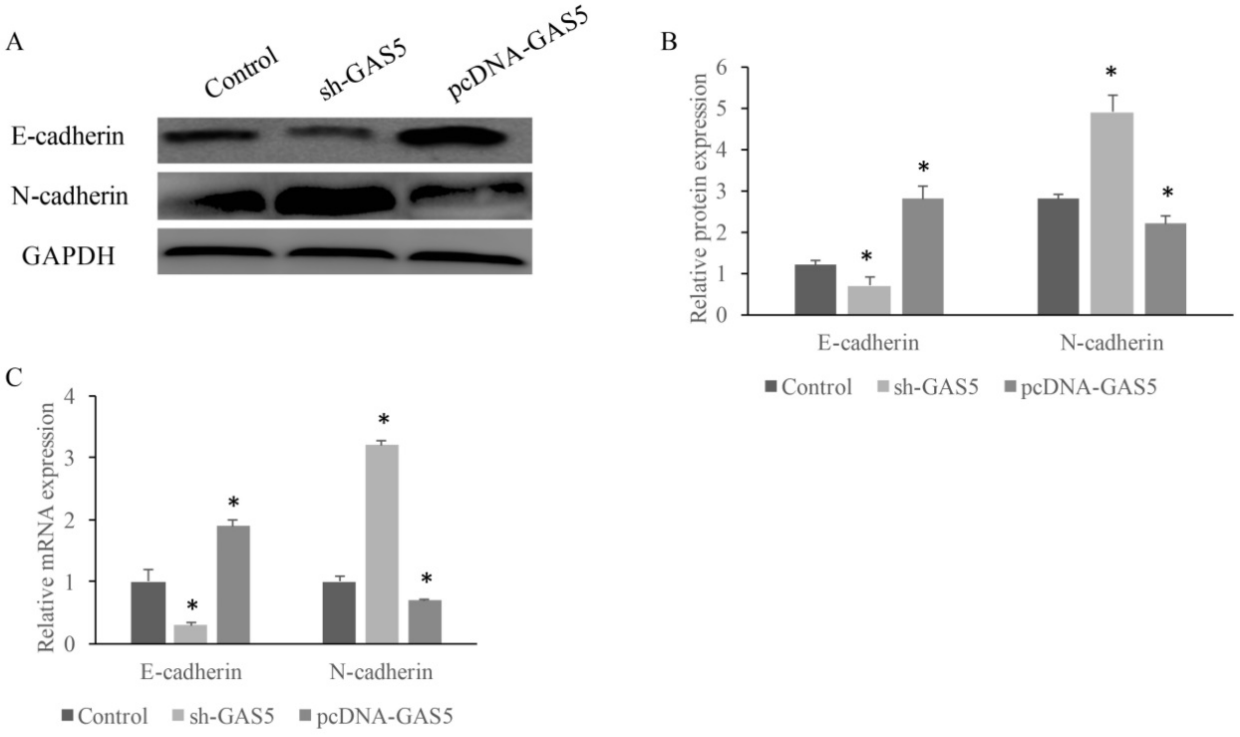
GAS5 markedly increased expression of E-cadherin and inhibited N-cadherin. (A) Western blot analysis of E-cadherin and N-cadherin expression; (B) quantification analysis of cadherin protein expression among SH-GAS5 and PCDNA-GAS5 cell lines; (C) real time PCR analysis of E-cadherin and N-cadherin expression. * P<0.05 compared with group Control. The results were achieved by conducting at least 3 independent experiments.

**Figure 5:**
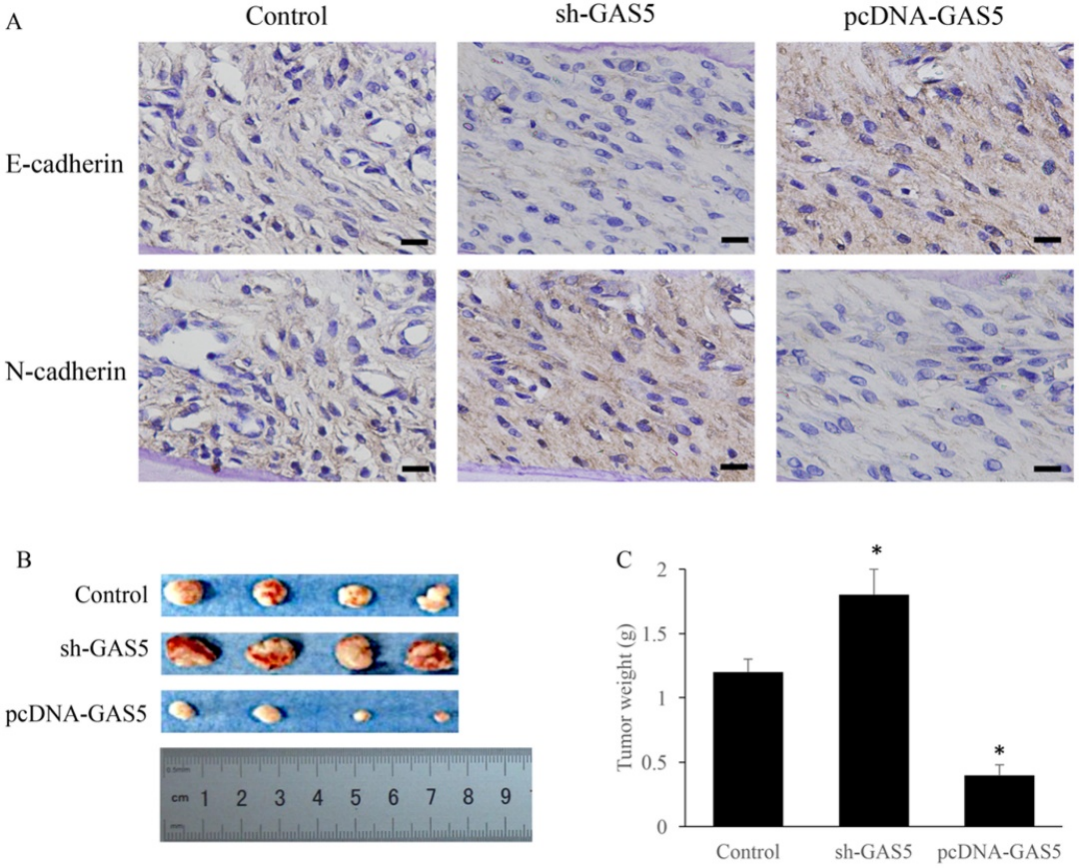
GAS5 markedly inhibited the growth of tumor in mice and induced EMT pathway. (A) Immunohistochemistry staining about E-cadherin and N-cadherin expression among sh-GAS5 and pcDNA-GAS5 cell lines, scale bar: 40 µm; (B) representative pictures of tumor among sh-GAS5 and pcDNA-GAS5 cell lines; (C) measurement of tumor weight. * P<0.05 compared with group Control. Four mice each group were used in this animal experiment. The results were achieved by conducting at least 3 independent experiments.

E-cadherin and N-cadherin were the biomarkers of EMT pathway, the E-cadherin and N-cadherin levels in knock-down or overexpressed H-460 were measured. E-cadherin expression increased nearly 2-folds and N-cadherin reduced markedly in GAS5 overexpressed group. However, sh-GAS5 inhibited the E-cadherin and increased the N-cadherin mRNA expression (Figure 4C). Western blot results indicated that the changes were correlated with mRNA level (Figure 4A-B). Immunohistochemical experiments showed that sh-GAS5 inhibited the expression of E-cadherin and increased the expression of N-cadherin, while pcDNA-GAS5 induced the expression of E-cadherin and reduced the expression of N-cadherin (Figure 5A). The tumor size of sh-GAS5 group was obviously enlarged compared with the tumors in control group and overexpression group (Figure 5B-C). Above all, the GAS5 indeed did suppress the growth of metastatic tumor cells *in vivo*.

### GAS5 inhibited chemotherapy sensitivity of lung cancer cells

**Figure 6:**
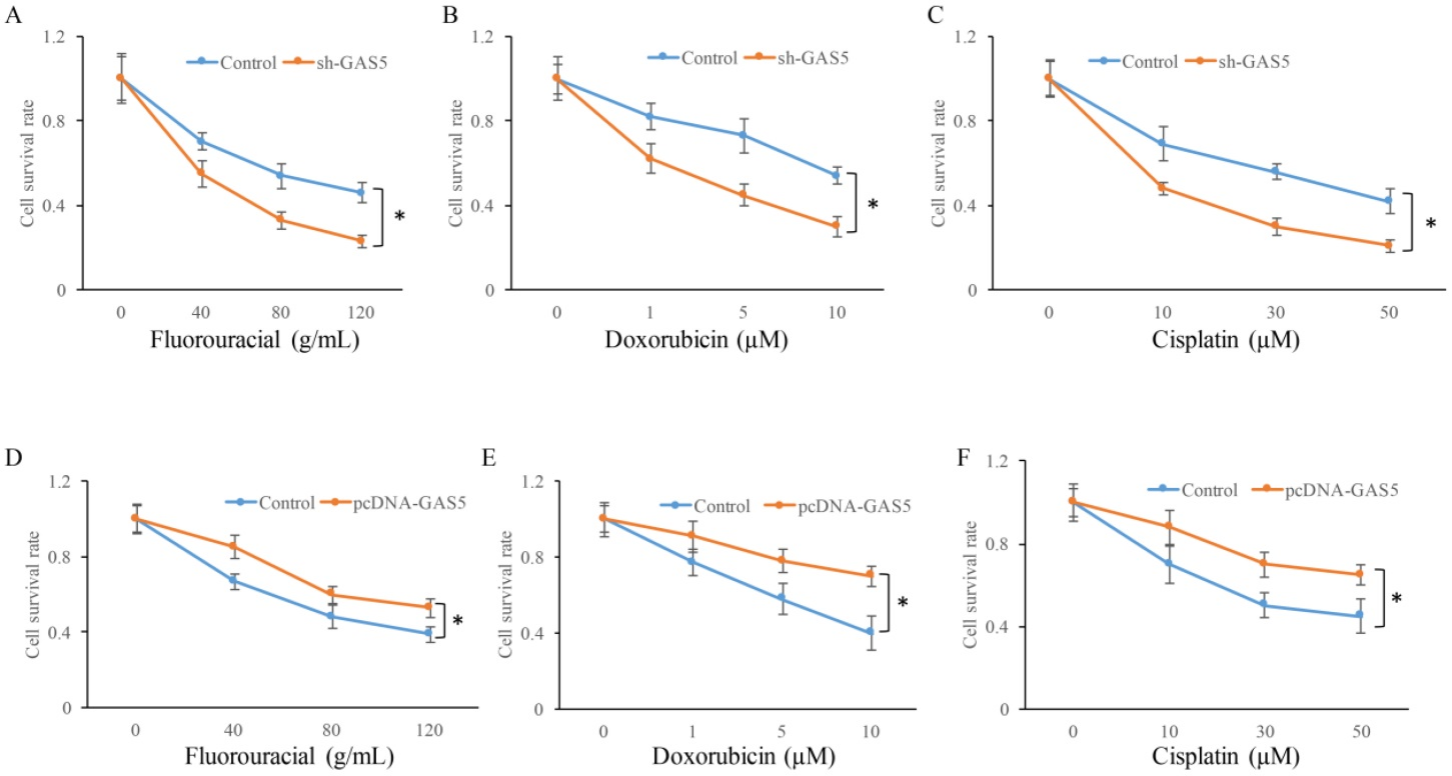
Influence of GAS5 on chemotherapy sensitivity of lung cancer cells. (A) Influence of GAS5 knockdown on cell survival after treatment with fluorouracial; (B) Influence of GAS5 knockdown on cell survival after treatment with doxorubicin; (C) Influence of GAS5 knockdown on cell survival after treatment with cisplatin; (D) Influence of GAS5 overexpression on cell survival after treatment with fluorouracial; (E) Influence of GAS5 overexpression on cell survival after treatment with doxorubicin; (F) Influence of GAS5 overexpression on cell survival after treatment with cisplatin. * P<0.05 compared with group Control. The results were achieved by conducting at least 3 independent experiments.

Whether the change of GAS5 expression could affect the chemotherapy sensitivity of lung cancer cells using three common chemotherapeutic molecular drugs was also observed. GAS5 knockdown in H-460 cells could significantly decrease cell survival in three different drug treatments compared to control (P<0.05) (Figure 6A, B and C). However, GAS5 overexpression could effectively promote cell survival in three different drug treatments compared to normal (P<0.05) (Figure 6D, E, and F). These results indicated that GAS5 were closely related to cell survival rate changes caused by drug treatments.

## Discussion

Numerous studies show that lung cancer cells proliferation and malignant could be influenced by abnormal transcription and expression of lncRNA genes. Some lncRNAs interfered with cell apoptosis and lead to excessive cell growth, thus causing malignant lung tumors. LncRNA UCA1 could bind to heterogeneous nuclear ribonucleoprotein 1 by competing with p27 gene, thus inhibiting the expression of p27 and promoting the proliferation of lung cancer cells. LncRNA CCAT2 can promote the proliferation of lung cancer cells by affecting Wnt signaling pathway, lncRNA PinX1 can block the growth of lung cancer cells in G0/G1 phase, thus inhibiting the proliferation of lung cancer and acting as anti-tumor effect 20. In this study, we found that GAS5 could promote cell apoptosis, and increase S phase ration and reduced G2 phase ratio.

Hypoxia inducible factor caused Ephrin-A3 to gather on the cell surface by increasing the expression of lncRNA EFNA3 21, which induced tumor cells infiltration into surrounding tissues from blood vessels, thus promoting the invasion and metastasis of cancer cells. LncRNA can also act as a tumor suppressor to regulate the invasion and metastasis of cancer. Liu et al confirmed that lncRNA NKILA was related to the NF-KB pathway and could be up-regulated by the inflammatory factors 22. In this study, GAS5 significantly inhibited cell migration and invasion. *In vivo* study showed that cancer proliferation was restricted and narrowed in size. More mechanism pathways should be explored about the roles of lncRNAs in cancer.

EMT refers to the biological process in which epithelial cells are transformed into mesenchymal phenotypic cells through specific procedures, and plays an important role in the process of cancer metastasis. Hu et al. used lncRNA chips to analyze the EMT process of cancer cells, and found that there were over 99 lncRNAs involved in the process, among which 4l played an important role in WNT signaling pathway by regulating target genes 23. Hou et al. 24 found that EMT could also be detected if lncRNA ROR was up-regulated. LncRNA ROR prevented ZEB2 from degradation, induced EMT expression, and thus promoted cell invasion and metastasis. Furthermore, *in vivo* studies confirmed that lncRNA-ROR could inhibit the growth of cancer cells and lung metastasis. Matouk et al. found that EMT were activated during the overexpression of lncRNA H19, which formed a positive feedback loop to promote the infiltration and metastasis of cancer 25. In addition, a large number of studies have found that lncRNA could also regulate the EMT process of cancer through its role in related signaling pathways.

Here, it is proved that GAS5 was closely related to cell apoptosis, and its down-regulated expression in cancer tissues predicts poor prognosis of lung cancer patients. Therefore, the transcription level of GAS5 can be regulated through the application of new targeted drugs or combination therapy to improve the prognosis of lung cancer patients. The study shed light on GAS5 as a novel therapeutic strategy.

In summary, this study has proved that GAS5 was closely related to epigenetic regulation of lung cancer. GAS5 could influence invasion and metastasis of lung cancer through EMT process, which shed light on the prospect for development as a therapeutic target for lung cancer.

## Authors' contributions

LZ, DZ and XP conceived and designed the experiments; TG, WC, YD, WL, YH, and JH performed the experiments, XP wrote the paper.

## Figures and Tables

**Table 1:** Primers information for qRT-PCR

Gene name	Primer sequence (5'-3')	
	Forward	Reverse
GAPDH	GTAGGCAAGCTGCGACGTGG	TGAACCTAAAACTGCTCTGA
E-cadherin	CACGCTGTGTCATCCAACGG	TGTAAGCGATGGCGGCATTGT
N-cadherin	CAGGAGCTGACCAGCCTCCAAC	TCAATTGCTGTTACGGTCATC

## Abbreviations

lncRNA GAS5): Long noncoding RNA GAS5
EMT): Epithelial-mesenchymal transition
qRT-PCR): Quantitative RT-PCR
GAPDH): Gglyceraldehyde-3-phosphate dehydrogenase
HRP): Horse radish peroxidase
FBS): fetal bovine serum
